# Detection of Chips on the Threaded Part of Cosmetic Glass Bottles

**DOI:** 10.3390/jimaging11030077

**Published:** 2025-03-04

**Authors:** Daiki Tomita, Yue Bao

**Affiliations:** Graduate School of Integrative Science and Engineering, Tokyo City University, Setagaya Campas, Tokyo 158-8557, Japan; bao@tcu.ac.jp

**Keywords:** image processing, chip inspection, glass bottles, infrared backlight, infrared camera

## Abstract

Recycled glass has been the focus of attention owing to its role in reducing plastic waste and further increasing the demand for glass containers. Cosmetics glass bottles require strict quality inspections because of the frequent handling, safety concerns, and other factors. During manufacturing, glass bottles sometimes develop chips on the top surface, rim, or screw threads of the bottle mouth. Conventionally, these chips are visually inspected by inspectors; however, this process is time consuming and prone to inaccuracies. To address these issues, automatic inspection using image processing has been explored. Existing methods, such as dynamic luminance value correction and ring-shaped inspection gates, have limitations: the former relies on visible light, which is strongly affected by natural light, and the latter acquires images directly from above, resulting in low accuracy in detecting chips on the lower part of screw threads. To overcome these challenges, this study proposes a method that combines infrared backlighting and image processing to determine the range of screw threads and detect chips accurately. Experiments were conducted in an experimental environment replicating an actual factory production line. The results confirmed that the detection accuracy of chipping was 99.6% for both good and defective bottles. This approach reduces equipment complexity compared to conventional methods while maintaining high inspection accuracy, contributing to the productivity and quality control of glass bottle manufacturing.

## 1. Introduction

### 1.1. Research Background

In recent years, Japanese cosmetic glass bottles have attracted attention in terms of improved manufacturing technology and environmental friendliness through the use of recycled glass [1], and the amount of glass bottles exported overseas is on the rise [2].

Glass bottles used as cosmetic bottles are treated as high-end products and are subject to strict quality inspections. In particular, the mouths of glass bottles are often equipped with screw threads for opening and closing the caps, and their complex shapes easily cause defects during production. In visual inspection, where defective products are judged by humans, research by Hida et al. has shown that different workers have different criteria for judgment and that long hours of work can cause a loss of accuracy [3]. This calls for the development of inspection equipment that uses image processing to improve productivity.

### 1.2. Related Work

To improve productivity, it is necessary to develop a detection device using image processing [1,2,3]. Many inspection methods using image processing have been proposed [4,5,6,7]. However, their targets are plastics and metals, and if the glass bottle is transparent, it cannot be inspected successfully due to its light-transmitting characteristics. In addition, glass bottle mouth inspection methods using image processing include a glass bottle mouth inspection method employing dynamic brightness value correction [8] and a glass bottle inspection method using ring-shaped inspection gates [9]. The former uses six cameras for side inspections and two cameras for top inspections to perform omnidirectional inspection. However, it is limited to inspecting simple-shaped glass bottles without screw threads, and the accuracy of flaw detection is low because of the use of natural light. The latter captures images from above but cannot inspect the entire screw-threaded portion effectively. To address these challenges, we propose improving the detection accuracy through the use of an infrared backlight and limiting the inspection area to critical regions.

## 2. Proposed Method

**Figure 1:**
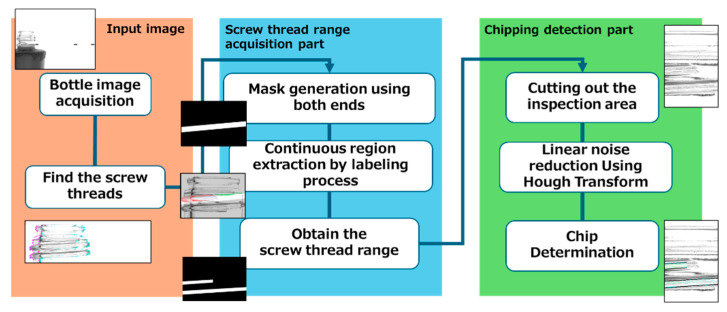
Overview of the proposed method.

The proposed method primarily focuses on obtaining a range of screw threads from captured images and detecting flaws. Figure 1 provides an overview of the proposed method. Each step is explained in detail in the following sections.

### 2.1. Capturing Bottle Images

**Figure 2:**
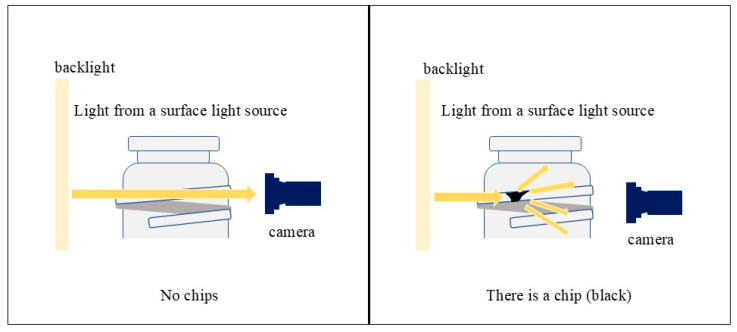
Overview of glass bottle photography.

An infrared surface light source and an infrared camera are employed for image capture. Since the glass bottles used in this study are transparent, light penetrates the smooth, undamaged surface when photographed under an infrared surface light source, resulting in a uniformly white appearance. However, the presence of chips or scratches causes diffuse reflection of light, making these areas appear darker. This optical property is utilized for defect detection. Figure 2 shows an overview of the glass bottle photography process, which simulates the way chips appear when infrared light is used. Additionally, a cut filter is applied to block ambient light, ensuring that inspections remain unaffected by external lighting conditions in the factory or inspection area.

### 2.2. Reasons for Limiting the Scope

During manufacturing, chipping is less likely to occur near the beginning and end of the screw threads and on the bottle surface because these areas are more exposed to air compared to the center and top of the screw threads, where the temperature distribution is more uniform during the cooling process [10]. Consequently, the possibility of chipping in these areas is significantly lower. To minimize false detections caused by noise, these areas are masked and excluded from the detection process.

### 2.3. Detection of the Screw Thread Endpoints

**Figure 3:**
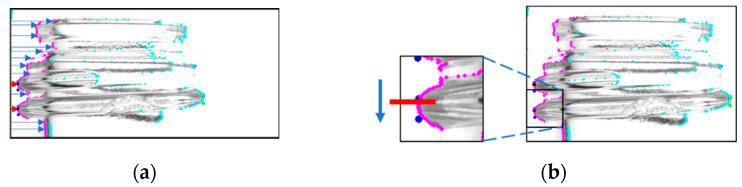
Process to search both ends of the screw threads. (A process that searches both ends (**a**) and a process that further searches the screw thread region (**b**)).

To obtain the screw threads, a search was first performed from the endpoints of the glass bottle. Figure 3a shows the scan starting from the upper-left corner of the captured image. The coordinates of the first pixel with the lowest luminance value, which corresponds to the outline of the glass bottle, were retained. The leftmost and second points from the left were retained as the endpoints of the screw threads based on the coordinate information of the outline. Subsequently, the top and bottom edges of the screw threads were determined by scanning a few pixels above and below the retained coordinates and selecting the two coordinates that fell within the specified pixel range. A similar scan was performed on the right side to locate the end of the screw thread. Figure 3b illustrates this process. The black dots indicate the ends of the screw threads, and the blue dots indicate the top and bottom coordinates determined during this process.

### 2.4. Mask Generation and Screw Thread Range Detection

**Figure 4:**
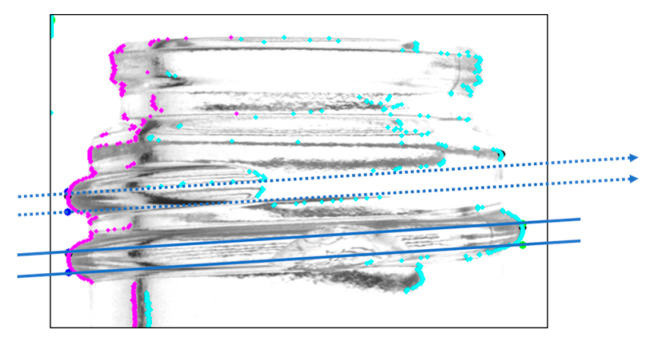
Screw thread range depicted when both endpoints are successfully identified (Solid line; for screw threads on both ends. Dotted line; for screw threads on only one side and complements by predicting).

The screw thread range was determined by connecting the coordinates obtained in the previous section. However, if a screw thread is cut in the middle, as illustrated in Figure 4, the coordinate information on one side is retained, while the coordinate information on the other side is unavailable. This makes it impossible to correctly determine the extent of the screw thread.

**Figure 5:**
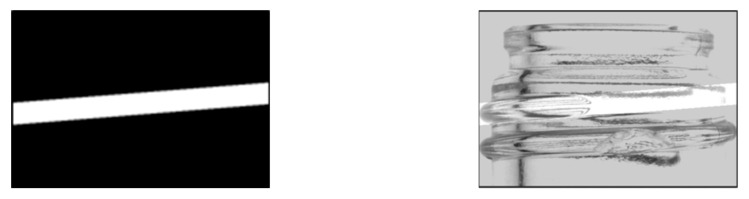
Generated mask image and the resulting composite image.

Therefore, a mask image was generated to accurately identify the screw thread missing on one side. As shown in Figure 5, the mask image was created with a thickness sufficient to cover the top and bottom of the screw thread. The endpoint of the screw thread was then located by detecting a shadow area that is continuous above and below the screw thread. This mask image was generated based on the slope obtained during the determination of the screw-thread range when both endpoints were successfully connected.

### 2.5. Image Processing

The image of the mouth of a cosmetic glass bottle often contains minute noise, which was removed using a bilateral filter [11]. This filter effectively eliminates microscopic noise while preserving image contours. In addition, contrast transformation was applied to enhance the distinction between light and dark areas, making scratches and non-scratches more discernible [12].

### 2.6. Screw Thread Range Detection Using Labeling

**Figure 6:**
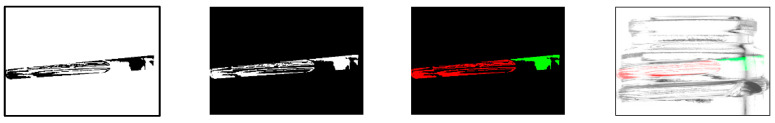
Labeling process.

For missing screw threads, the thread range was determined by identifying contiguous areas through the labeling process. This process used OpenCV’s “ConnectedComponentsWithStats” function [13]. As shown in Figure 6, the generated mask image was combined with the contrast-enhanced image to generate an image containing only the screw thread portion. Labeling was then performed by tracking the shadows of the upper and lower portions of the screw threads to determine their full extent.

**Figure 7:**
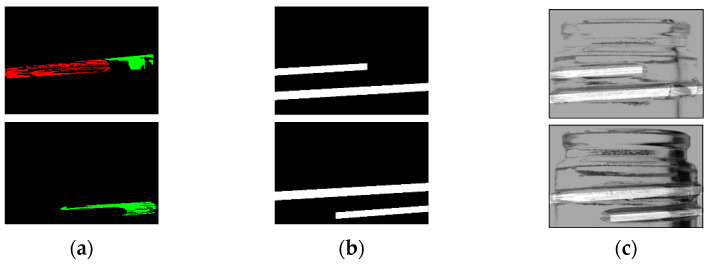
Generated mask and synthesized images. (**a**) Label image, (**b**) mask image, (**c**) composite image.

Based on the labeling information obtained, a mask image was generated to match the screw thread range. The generated mask image and the bottle image synthesized using the mask are shown in Figure 7. For example, the red label information is used to identify the screw thread regions contiguous from the left, and the green label information is used to identify the screw thread regions contiguous from the right.

### 2.7. Cutting Out the Inspection Area

**Figure 8:**
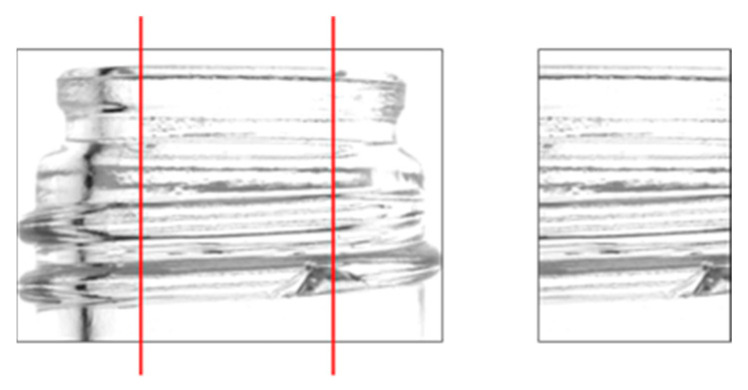
Cutout portion of the bottle image used for the detection. (The image to be cropped and retrieved with the red line is on the left).

Chipping detection performed on the entire bottle image can be significantly affected by shadows in areas not visible in the front view. To minimize the influence of these shadows and improve chip detection, only the center portion of the bottle image was cropped using the coordinates of the two endpoints identified in Section 2.3. An example of this cropping method is shown in Figure 8. Because one image was acquired by the left camera, two images by the center camera, and one image by the right camera for flaw detection, it was possible to perform 180° flaw detection even when only the center portion of the image was used.

### 2.8. Mask Generation Using Hough Transform

**Figure 9:**
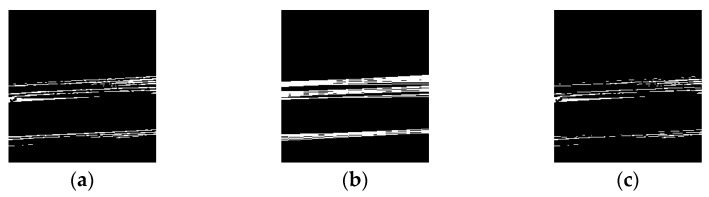
Hough transform process. (**a**) Hough transforms for each label, (**b**) generated mask image, (**c**) composite image.

As described in the previous sections, an image containing only the screw threads was obtained; however, it included significant linear noise owing to the shadows cast by the screw threads. To remove this noise, linear line detection using Hough transform processing was performed [14,15]. Labels identified as straight lines are removed by generating a mask to exclude them from the detection target. Figure 9 illustrates the mask generation process.

### 2.9. Chip Detection

**Figure 10:**
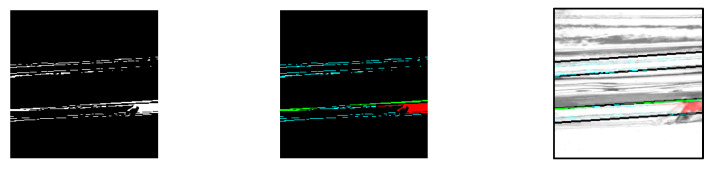
Process for extracting missing parts.

From the generated images, chips were detected based on the area, width, and height of each label. The labels are listed in Table 1. The chip detection process is illustrated in Figure 10.

## 3. Experiments

Two experiments were conducted to verify the effectiveness of the proposed method. In Experiment 1, one good bottle and two defective bottles were rotated by 30°, and images were acquired 10 times at each angle to confirm that 180-degree side detection was achievable. In Experiment 2, the orientation of the glass bottles was adjusted to match that of an actual factory production line. A total of 100 good bottles were placed on the conveyor four times, while 29 defective bottles were placed on the conveyor twice.

### 3.1. Experimental Environment

**Figure 11:**
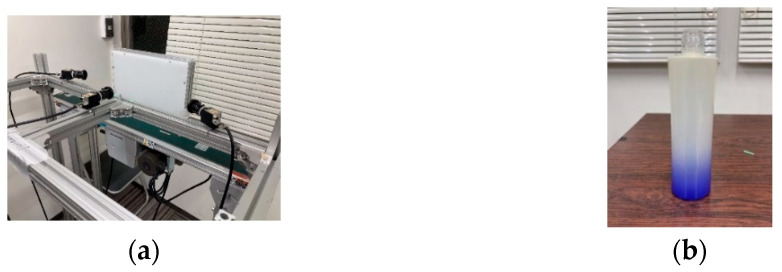
Experimental environment. (**a**) Experimental setup and (**b**) glass bottle used in the study.

**Figure 12:**
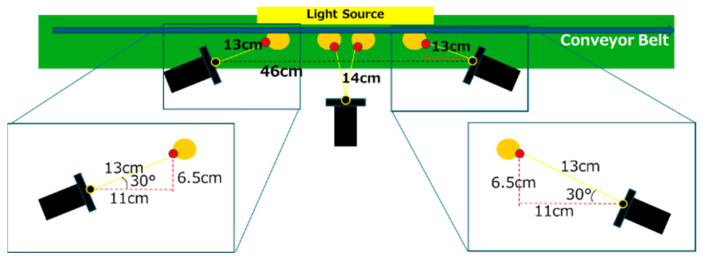
Schematic drawing of the experimental environment.

Figure 11 illustrates the experimental environment and the glass bottles used in the study. Table 2 lists the components and their specifications for the experimental setup. In addition, Figure 12 provides a detailed diagram of the camera arrangement. A constant voltage controller was utilized for image capture, with the light intensity set to its maximum level and maintained at a constant value. A cut filter was applied to block wavelengths other than 850 nm, effectively eliminating ambient light interference.

### 3.2. Taking Bottle Images

**Figure 13:**
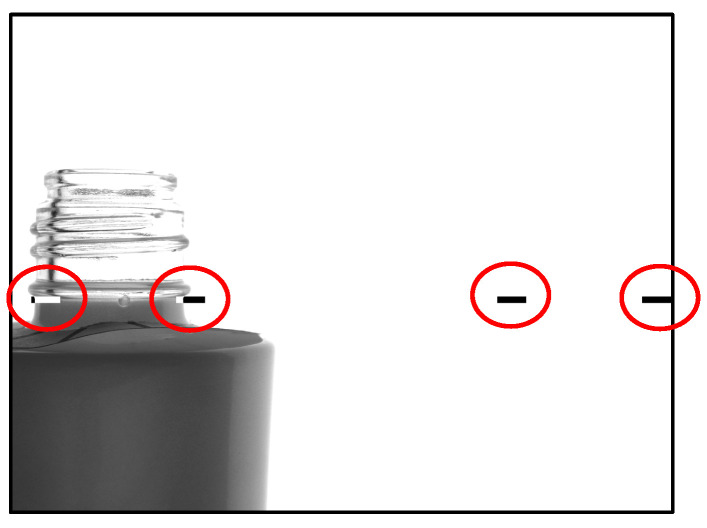
How to take bottle images.

The bottles transported onto the conveyor appear as shown in Figure 13 when captured by the camera. As the bottles move along the conveyor, images are taken based on two reference markers (indicated by red circles), and the relevant regions are extracted. In this example, the central camera is depicted, capturing two images—one from each side of the bottle.

### 3.3. Detection Experiment on the Side of the Bottle

**Figure 14:**
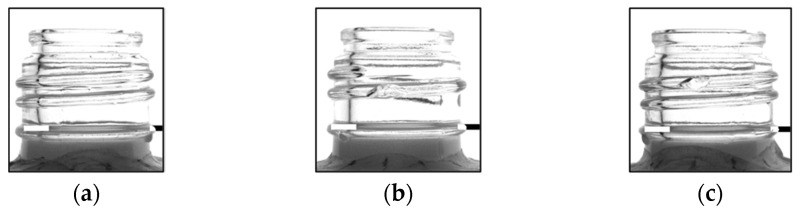
Examples of good bottles and defective bottles presenting chips. (**a**) Good, (**b**) D1, (**c**) D2.

In this study, a detection of 180° was achieved. To verify the detection rate of the half-face (180°) of the bottles using this method, one good and two defective bottles (D1 and D2) were placed on the conveyor belt, and their images were acquired 10 times at 30° intervals, resulting in a total of 210 images, to ensure that the method could accurately inspect them. One bottle with chips on both the top and bottom of the screw threads was selected as the sample to confirm that there were no issues in detecting flaws during the acquisition of the screw threads. Figure 14 shows images of one good and two defective bottles acquired using an infrared camera.

### 3.4. Experimental Results (Experiment on the Side of the Bottle)

**Figure 15:**
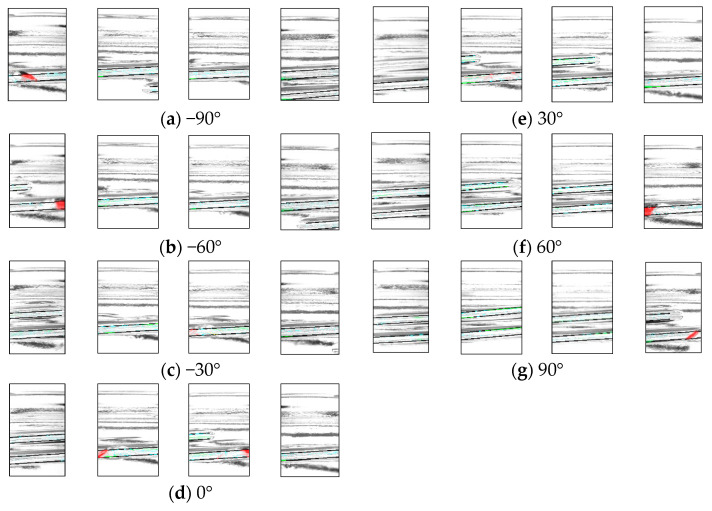
Experimental results show images of bottle defective 1 at different angles.

Figure 15 shows a portion of the resulting image, with the chipped areas indicated in red, indicating successful detection. The image shows the D1 rotated by 30°. In addition, Table 3, Table 4 and Table 5 present a breakdown of the experimental results. Table 3 presents the results of inspecting good bottles 10 times per angle, Table 4 shows the results of inspecting D1 10 times per angle, and Table 5 shows the results of inspecting D2 10 times per angle.

### 3.5. Discussion of Results (Experiment on the Side of the Bottle)

**Figure 16:**
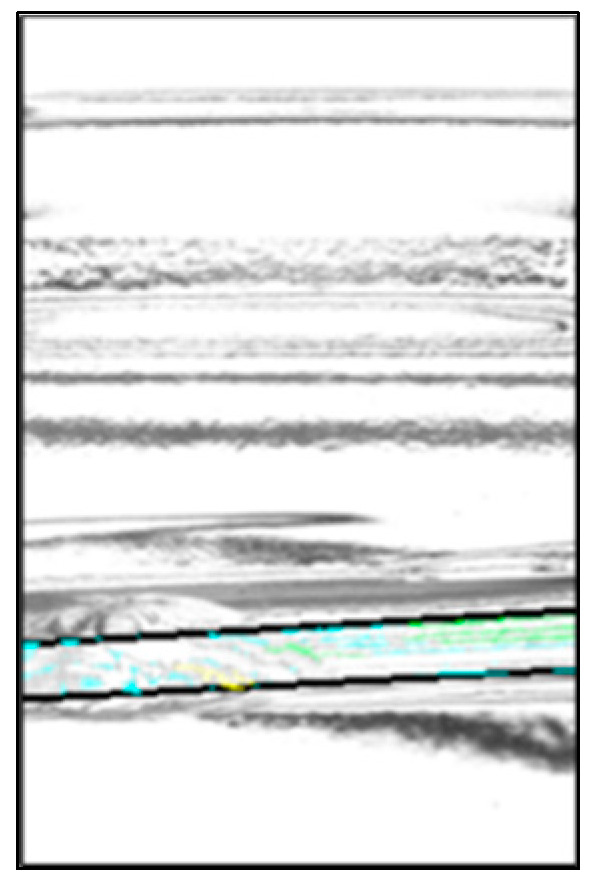
Example of the false detection.

The chip detection rates were 98.6% for defective products, 97.1% for D1, and 100% for D2. In the experiment involving good bottles, 100% were correctly identified as good. The overall inspection accuracy rate for the entire set, including both good and defective bottles, was 99.0%. The cameras were able to detect the chips even when the bottles were rotated by 30°. This confirmed that the three-camera system could effectively detect chips on half of the bottles. Figure 16 shows an image of an inspection result that was erroneously detected as a defective product as an example of a false detection. It was found that the chips were determined as noise.

### 3.6. Experiments Simulating a Production Plant

In an actual factory production line, glass bottles flow on a conveyor at a specific angle because of the product labeling process. To simulate this, we conducted an experiment in which glass bottles were aligned in the same direction as they would be in a production plant. The sample set for this experiment included 100 good bottles and 29 defective bottles. In the experiment, good bottles were placed on the conveyor four times each to acquire 400 images, and defective bottles were placed twice to acquire 58 images.

### 3.7. Experimental Results (Experiments Simulating a Production Plant)

**Figure 17:**
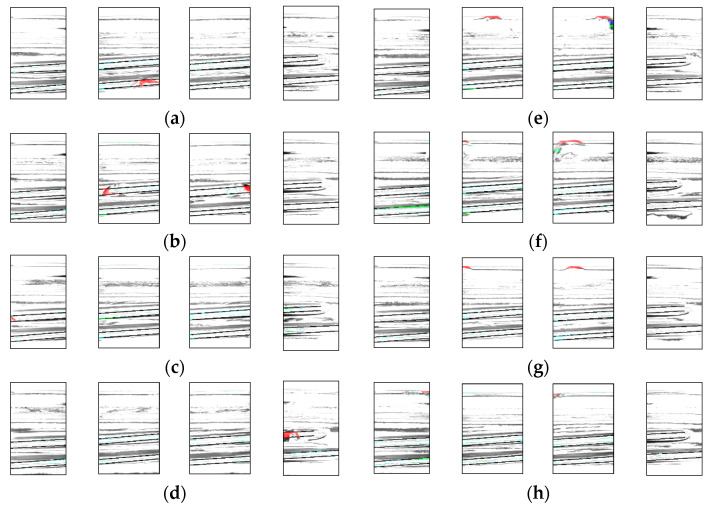
Example of detection. Result for (**a**) chip1, (**b**) chip2, (**c**) chip3, (**d**) chip 4, (**e**) chip5, (**f**) chip6, (**g**) chip7, (**h**) chip8.

Figure 17 shows an example of a resulting image from a normal inspection by each camera. The areas shown in red indicate the chips that should be detected using this method. The image also displays a portion of the 29 bottles containing chips. A breakdown of the experimental results is presented in Table 6.

### 3.8. Discussion of Results (Experiments Simulating a Production Plant)

**Figure 18:**
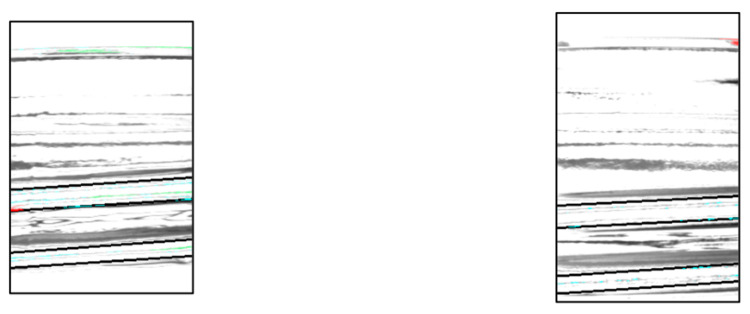
Example of false positive.

As a result of the experiment, 3 false positives were detected at the screw threads and 19 false negatives at the top surface, resulting in an inspection accuracy of 94.5%. For defective products, no false positives were detected, achieving an inspection accuracy of 100%. Figure 18 shows examples of false-positive detection for a good bottle.

### 3.9. Additional Experiments Based on the Experimental Results

**Figure 19:**
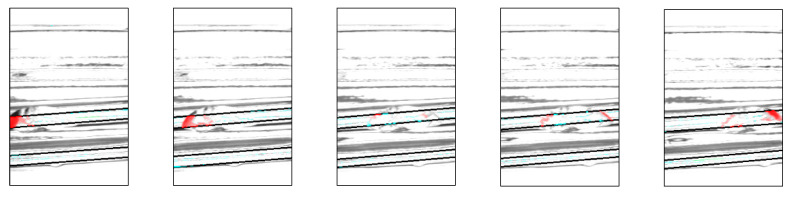
Example for minute angle changes.

Based on the results of Experiments 1 and 2, additional experiments were conducted to study the reflection of the chips at different angles using a single camera. Examples of chip detection obtained by applying minute angle changes are shown in Figure 19.

## 4. Proposed Enhancements and Results

### 4.1. Binarization Process According to the Curved Surface of the Bottle

**Figure 20:**
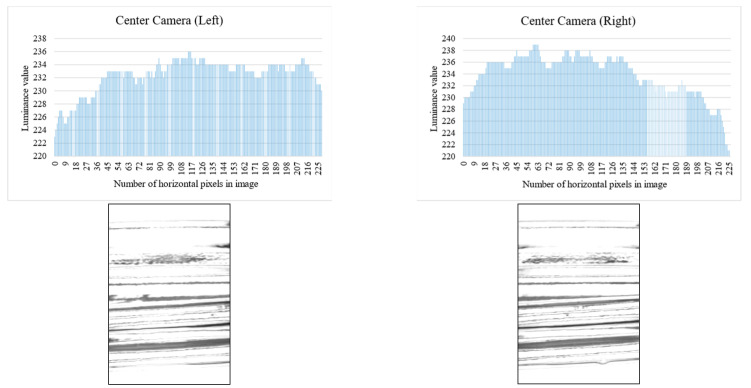
Relationship between inspection image and luminance value (center camera).

**Figure 21:**
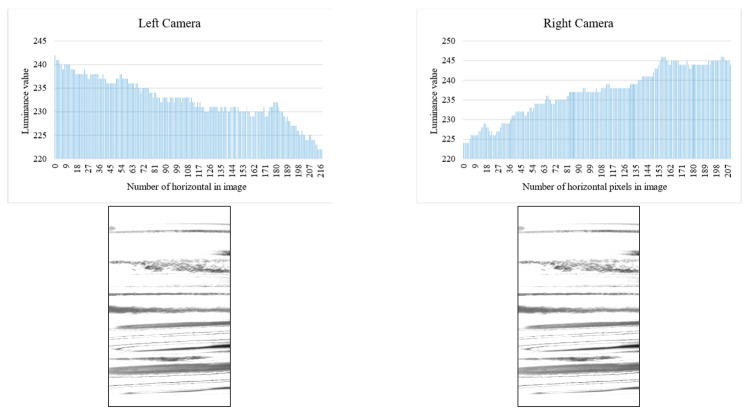
Relationship between inspection image and luminance value (left and right camera).

Binarization is performed on the contrast-adjusted image by applying a labeling process for chip detection. The glass bottle being inspected has a curved surface. When light from a surface light source strikes a curved surface, it is reflected, causing an increase in refraction and a decrease in luminance value. Figure 20 and Figure 21 illustrate the relationship between the average luminance values and the curved surface.

**Figure 22:**
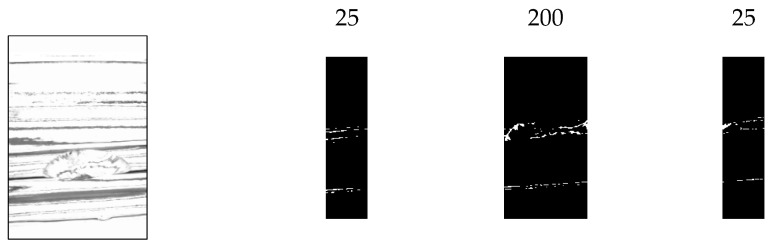
Threshold change process.

In addition, when images of bottles with chips were acquired from multiple angles, it was observed that when the chips were in front of the image, the reflection was weak, making it difficult to identify the chips. Based on this observation, the inspection image was divided vertically, as shown in Figure 22, and different binarization thresholds were applied to each section.

### 4.2. Subtractive Processing of the Top Surface Area

**Figure 23:**
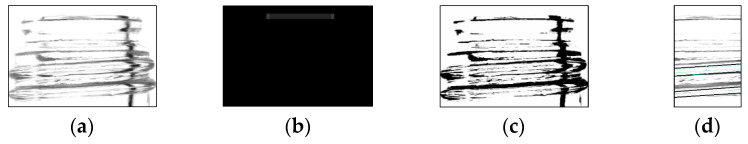
Pixel value addition process. (**a**) Photographic image, (**b**) added image, (**c**) enhanced image, and (**d**) result image.

In Experiment 2, false detections due to noise were observed on the top surface area of the bottles. To address this, we propose a method that involves adding a certain pixel value to the top surface area. In the proposed method, the contrast was adjusted to emphasize the chips. However, this process also emphasized noise along with the chips. As shown in Figure 23, by adding a certain pixel value to the top surface of the entire inspection area, it may be possible to emphasize the chips while reducing the noise.

### 4.3. Experimental Setup

In the third experiment, the proposed method was added, and the experiment was conducted in the same manner as in Experiment 2, using images acquired in Experiment 2 for 100 good products four times and 29 defective products.

### 4.4. Experimental Results

The experimental results are shown in Table 7.

### 4.5. Discussion of Results

**Figure 24:**
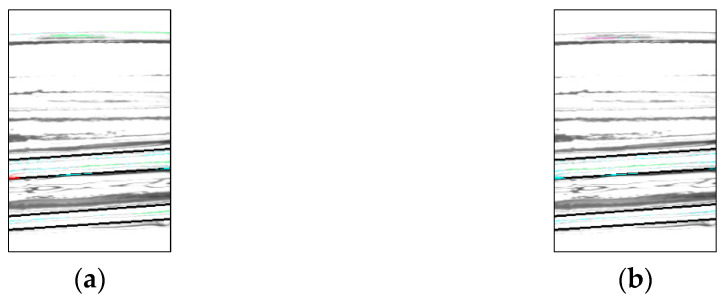
Comparison with Experiment 2 (screw thread). (**a**) Experiment 2 and (**b**) Experiment 3.

**Figure 25:**
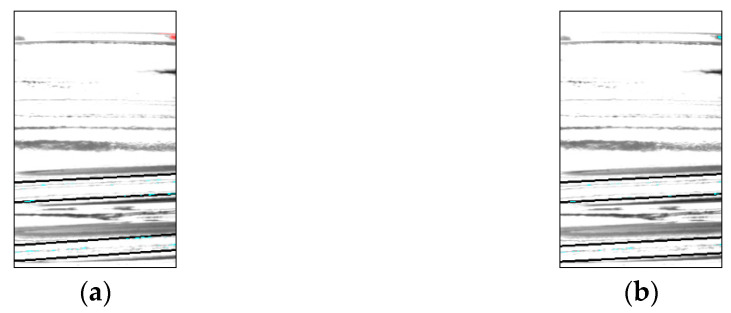
Comparison with Experiment 2 (top). (**a**) Experiment 2 and (**b**) Experiment 3.

**Figure 26:**
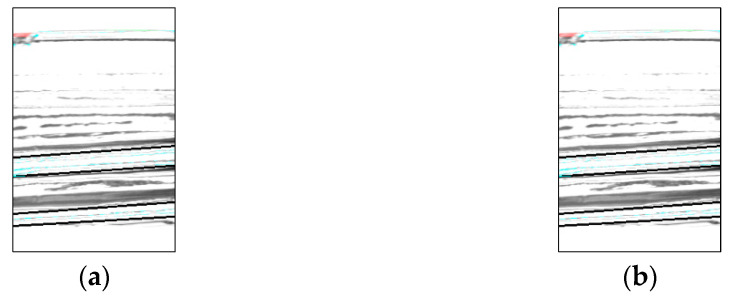
Comparison with Experiment 2 (defect bottle). (**a**) Experiment 2 and (**b**) Experiment 3.

Table 7 and Figure 24 and Figure 25 show the additional proposed method improved inspection accuracy for good bottles. However, for defective bottles, two false positives occurred, which did not occur in Experiment 2. An example of a false-positive detection is shown in Figure 26. The accuracy of the inspection was reduced because the addition of pixels caused some pixels to be missing from the image, making it difficult to accurately identify the missing areas.

### 4.6. Comparison with Conventional Method

Table 8 and Table 9 summarize the inspection results of a previous study [4] and those of the proposed method.

The accuracy of the inspection was comparable to that of previous studies. However, one prior study used six cameras for the sides of the bottle and three cameras for the top surface to achieve 360° detection [4]. In this study, although the bottle-half surface was 180°, the side and top surfaces could be inspected with only three cameras. We believe that this approach will contribute to reducing the weight of the equipment used.

## 5. Conclusions

In this study, we aimed to improve inspection accuracy by using an infrared backlight and limiting the inspection area. The results of Experiment 1 showed that good and defective bottles were correctly detected when rotated every 30°, with sufficient accuracy to inspect the sides of the bottles. Experiment 2 demonstrated inspection accuracies for good and defective bottles of 94.5% and 100%, respectively. The results of Experiments 1 and 2 confirm that the reflection of scratches depends on the refractive index and reflectivity of the curved surface area of the glass bottle. Based on these findings, we proposed methods to adjust the binarization threshold and to add specific pixel values to the top surface to improve inspection accuracy in Experiment 3. The results of Experiment 3 confirmed the effectiveness of these methods, achieving a 100% detection rate for good bottles and an inspection accuracy of 96.5% for defective bottles. The parameters of the proposed method exhibit a trade-off between good and defective bottles, and future work will focus on optimizing these parameters to enhance detection accuracy. In addition, we would like to prepare two experimental apparatuses to check the accuracy of 360° inspection and to see if they can be used for different types of glass bottles. Finally, it was found that the method proposed in this study has the potential to improve productivity in glass bottle production and advance the industry.

## Figures and Tables

**Table 1:** Criteria for chip detection and corresponding label colors.

Detection Criterium	Label Color
Area < Minimum area threshold	Light Blue
Area > Maximum area threshold	Pink
Spans the entire width of the inspection area	Blue
Width/Height > Set threshold	Green
Width/Area > Set threshold	Yellow
Other (Chipping, cracks)	Red

**Table 2:** Equipment used in the experiment.

Component Parts	Specification	
Camera	Model number	VCXU-32M
	Resolution	2048×1536
Lens	Model number	VS-1218VM
	Focal Length	12 mm
	Angle of view (1/1.8″)	25°×32.6°
Filter	Model number	ZOMEI IR850
Infrared backlight	Model number	IFD-300/200IR-850
Light source power supply	Model number	IWDV-300S-24

**Table 3:** Experimental results for the good bottle.

Detection	−90°	−60°	−30°	0°	30°	60°	90°
Positive	10	10	10	10	10	10	10
False	0	0	0	0	0	0	0

**Table 4:** Experimental results for D1.

Detection	−90°	−60°	−30°	0°	30°	60°	90°
Positive	10	10	8	10	10	10	10
False	0	0	2	0	0	0	0

**Table 5:** Experimental results for D2.

Detection	−90°	−60°	−30°	0°	30°	60°	90°
Positive	10	10	10	10	10	10	10
False	0	0	0	0	0	0	0

**Table 6:** Experimental results.

		Good	Defective
Positive		378	58
False positive	Screw thread	3	0
	Top	19	0
Detection rate		94.5%	100%

**Table 7:** Experimental results.

		Good	Defective
Positive		400	56
False positive	Screw thread	0	0
	Top	0	2
Detection rate		100%	96.2%

**Table 8:** Detection accuracy of the conventional method [4].

	Sample	Recognized	Recall
Good bottles	100	98	98%
Defect bottles	100	98	98%

**Table 9:** Detection accuracy of the proposed method.

	Sample	Recognized	Recall
Good bottles	400	400	100%
Defect bottles	58	56	96.5%

## Data Availability

The raw data supporting the conclusions of this article will be made available by the authors on request.
